# Effects of *Saccharomyces cerevisiae*-lactic acid bacteria cocultured maize silage on dairy cows performance and *in vitro* rumen fermentation

**DOI:** 10.3389/fmicb.2026.1753173

**Published:** 2026-01-22

**Authors:** Qingqing Chen, Zixin Liu, Chuanshe Zhou, Zhiming Zhong, Jian Wu, Aoyu Jiang, Hai Yang, Zhiliang Tan, Bernard Adubwa Lukuyu, Jinhe Kang

**Affiliations:** 1 State Key Laboratory of Forage Breeding-by-Design and Utilization, National Engineering Laboratory for Pollution Control and Waste Utilization in Livestock and Poultry Production, and Hunan Provincial Key Laboratory of Animals Nutritional Physiology and Metabolic Process, Institute of Subtropical Agriculture, Chinese Academy of Sciences, Changsha, Hunan, China; 2 Institute of Geographic Sciences and Resources, Chinese Academy of Sciences, Beijing, China; 3 Hunan Key Laboratory for Conservation and Utilization of Biological Resources in the Nanyue Mountainous Region, Yuelushan Laboratory, Changsha, Hunan, China; 4 Animal Nutrition and Feeds, International Livestock Research Institute, Nairobi, Kenya

**Keywords:** fermentation quality, *in vitro* fermentation, microbial additives, milk composition, whole-plant maize

## Abstract

**Introduction:**

Microbial additives can improve silage quality in lowland areas. However, *Saccharomyces cerevisiae* and Lactic Acid Bacteriacan efficacy on whole-plant maize silage under Tibet’s hypoxic and cold environment, have not been explored.

**Methods:**

In this experiment, whole corn plants cultivated in Dazi District, Lhasa City, Xizang (Tibet) Autonomous Region, were selected as silage raw materials. The treatment group was added 0.5 kg of microbial additives per ton of silage. The addition levels for both *Saccharomyces cerevisiae* and Lactic Acid Bacteria were ≥ 1 × 107 CFU·g-1 FM). The quality of silage and its *in vitro* fermentation characteristics were determined on 0, 30 and 60 days of fermentation, respectively. Subsequently, dairy cows were fed with silage after 60 days of fermentation to evaluate milk production and milk quality.

**Results:**

The results indicated that the lactic acid content in the treatment group was increased significantly on 30 and 60 days of fermentation (*p* < 0.05). In addition to Simpson’s index, alpha diversity was significantly affected by the fermentation day × treatment interaction (*p* < 0.05). At 60 days of fermentation, the abundance of *Firmicutes phylum* in the treatment group was significantly higher than that in the control group (*p* < 0.05). The abundance of genera such as *Acetobacter* and *Latilactobacillus* was significantly decreased (*p* < 0.05), while the abundance of the genus *Weissella* was significantly increased (*p* < 0.05). Dairy cows were fed 60-day maize silage, the milk protein content and total solid content in the treatment group were significantly higher than that in the control group (*p* < 0.05). The levels of dry matter degradation rate, ammonia nitrogen and total volatile fatty acids in the *in vitro* fermentation of maize silage in the treatment group on the 60th day of fermentation were significantly higher than that in the control group (*p* < 0.05).

**Conclusion:**

In Xizang (Lhasa, China), the addition of microbial additives has significantly improved the quality and nutritional value of whole corn silage plants and enhanced the milk quality of local dairy cows. This provides a theoretical basis for the application of microbial additives from the Qinghai-Tibet Plateau to agricultural crops.

## 1 Introduction

Xizang’s extreme climatic conditions, characterized by prolonged frost periods and limited frost-free seasons, result in an extended dry non-growing forage season lasting more than 7 months each year. This leads to significant feed shortages during the winter and spring months, intensifying the imbalance between forage availability and livestock demand. Consequently, livestock producers are frequently compelled to cull animals prematurely due to inadequate feed resources (Liu et al., 2024). Silage is generally considered an effective method for preserving green fodder; however, such fermentation is challenging at low temperatures, making it prone to spoilage. Therefore, in high-altitude cold regions where year-round adequate feeding of ruminant livestock is necessary, enhancing the fermentation of silage crops is crucial for improving the nutritional value, preservation, and storage of green fodder (Jiang et al., 2023; Muck et al., 2018). Although microbial additives have been studied in temperate regions, their efficacy on whole-plant maize silage under Xizang hypoxic and cold environment, have not been explored. *Saccharomyces cerevisiae* and lactic acid bacteria are essential microorganisms found in the bodies of humans and animals, and have significant applications in food, healthcare, agriculture, and medicine (Liao et al., 2022). *Saccharomyces cerevisiae* exhibits strong adaptability to high acidity, a wide range of temperatures, and elevated bile salt concentrations. It is rich in nutrients, including minerals and proteins, which can be incorporated into animal feed. This incorporation utilizes sugars in silage, promoting the reproduction of beneficial microorganisms and increasing the protein content of the feed. Additionally, it can inhibit the production of aflatoxin B1 and generate ethanol and other compounds, resulting in fermented silage with a distinct wine flavor that enhances the palatability to animals (Fouad et al., 2019; Xu et al., 2019). Lactic acid bacteria can inhibit the growth of harmful microorganisms and reduce the loss of crude protein and water-soluble carbohydrates, thereby preserving more nutrients and creating an acidic environment conducive to fermentation (Aguirre-Garcia et al., 2024). Studies have demonstrated that supplementing rations with *Saccharomyces cerevisiae* additives can enhance the synthesis of rumen acetic acid, a key precursor in milk fat production, thereby improving dairy cows’ milk fat content, milk production, and overall productivity (Garnsworthy et al., 2025). Research indicates that the dominant bacterial genera in high-altitude pasture grasses are *Lactobacillus brevis* and *Lactobacillus plantarum*, which rapidly produce lactic acid and cause a decrease in pH (Bao et al., 2023; Ding et al., 2020). Zhou et al. (2019) reported that *Saccharomyces cerevisiae* increased the concentration of crude protein in silage. In contrast, lactic acid bacteria effectively utilize the water-soluble carbohydrates (WSC) in the silage material to generate organic acids, reduce the pH of the silage, and increase the concentrations of lactic acid (LA), acetic acid (AA), propionic acid (PA), and butyric acid (BA). The present study hypothesized that microbial additives can reduce the likelihood of spoilage and improve the quality of silage during storage. Furthermore, it aims to enhance the nutritional value of silage and promote more efficient utilization of available feed resources by ruminants in the feed-deficient in Xizang (Lhasa, China) region. This approach may also help alleviate seasonal dietary shortages commonly observed during winter and spring in the Xizang plateau. However, considering that Xizang herders regard cows as sacred animals, only limited sampling (e.g., milk samples) may be feasible, and more invasive procedures such as collecting rumen fluid are often impossible. Therefore, *in vitro* rumen fermentation was selected as the method to further evaluate the impact of maize silage on ruminal fermentation performance.

## 2 Materials and methods

### 2.1 Silage wrapping treatment

#### 2.1.1 Silage materials and methods

The experiment was carried out from October 18, 2023, to December 16, 2023, at the Golden Wheat Ears Breeding Specialized Cooperative located in Dazhi District, Lhasa, Xizang (Latitude: 29°84″N, Longitude: 91°57″E, Altitude: 3,600 m), with the objective of evaluating the fermentation performance of silage whole-plant maize silage prepared by film-wrapping. The climate of the site is a highland temperate semi-arid monsoon climate. The silage culture FW102 was provided by Shenzhen Saiyiyang Biotechnology Co., Ltd., the main ingredients included soybean meal 25%, rice bran 25%, corn germ meal 20%, molasses 10%, compound probiotics (*Saccharomyces cerevisiae*: lactic acid bacteria (LAB) = 5:5) 20%, with an effective count of viable bacteria of ≥ 1 × 10^7^ CFU·g^−1^ FM. The lactic acid bacterial strain belongs to the species *Lactobacillus plantarum*. The whole-plant maize variety used was Golden Apple 618, harvested in October 2023. The maize was in the milk and wax ripening stages, mowed, and cut into small segments of 2–3 cm, then thoroughly mixed with the microbial additives. After thorough mixing, the material was wrapped and compacted into bales using a wrapping machine, with each bale weighing 500 kg. The experiment was divided into two groups: the control group (Control group, Con), which received no microbial additives, and the treatment group (Treatment group, Treat), which had 0.5 kg of microbial additive per ton of whole maize plant, with no restrictions on the moisture content of the whole maize plant. Each group consisted of four replicates, with one bale per replicate. New silage bales were opened on the 0th, 30th, and 60th days of silage, respectively, to collect silage samples. The collected whole-plant maize samples were analyzed for their silage quality, microbial diversity, and *in vitro* rumen fermentation characteristics.

#### 2.1.2 Silage sample collection and analysis

On days 0, 30 and 60, the top 20 cm of each silage bale was discarded; thereafter, four replicate bales per treatment were opened. The contents were collected and divided into three subsamples.

The first part involved taking 800 g of the sample for the determination of nutritional indicators. The sample was dried in an oven at 65 °C for 48 h until a constant weight, then crushed and sieved through a 40-mesh sieve before being sealed and stored. We measured dry matter (DM) and ash content according to AOAC (Horwitz and AOAC International, 2006). Ash content was determined by combustion in a muffle furnace at (550 ± 20) °C for 3 h. Crude fat (EE) content was assessed using a Soxhlet fat extractor (Gerhardt). Kjeldahl nitrogen determination was performed using a Kjeldahl nitrogen analyzer (model: Fibertec™ 8000, serial number: 178811). Total nitrogen (TN) was determined according to the Kjeldahl procedure, and the crude protein (CP) content was calculated by multiplying the TN by 6.25 (Krishnamoorthy et al., 1982). The method of Van Soest et al. (1991) was employed for the determination of neutral detergent fiber (NDF) and acid detergent fiber (ADF).

In the second part of the study, 50 g of samples were collected to prepare leachate for the determination of fermentation indices. From each group after silage, 20 g of samples were taken and added to 180 mL of Ringer’s solution. The mixture was stirred for 30 min and then filtered through 4 layers of gauze to obtain the leachate, which was immediately used to measure the pH using a pH meter (PHS-3C, Shanghai LeiMagnet Scientific Instruments Co., Ltd., Shanghai, China). The extract was collected in a 15 mL centrifuge tube and stored in a refrigerator at −80 °C for subsequent determination of fermentation quality indices. The extracts were thawed and centrifuged, and the lactic acid content (LA) was measured using the Taylor colorimetric method (Taylor, 1996). The NH_3_-N content was determined by the phenol-hypochlorite method (Broderick and Kang, 1980). A gas chromatograph (Agilent 7890 A, Agilent Inc., Palo Alto, California, United States) equipped with a flame ionization detector and a capillary column (HP-INNOWAX 19091 N-133 [30.0 m × 0.25 mm × 0.25 μm]) was employed to analyze the concentrations of lactic acid and volatile fatty acids (VFAs), including acetate, propionate, and butyrate. During sample injection, a volume of 1 μL was used, including the internal standard, with nitrogen as the carrier gas. The temperature was increased from 60 °C to 200 °C at a rate of 20 °C/min and then held at 200 °C for 3 min. The inlet and detector temperatures were set at 250 °C and 300 °C, respectively, with a split ratio of 10:1. The flow rate of the hydrogen carrier gas was maintained at 25 mL/min.

In the third part of the study, 25-grams subsamples were collected for microbiological analyses. Fresh material was taken from the internal, anaerobic portion of the maize silage, placed in sterile, enzyme-free centrifuge tubes, and immediately stored at −80 °C until analysis. The silage samples were homogenized after thawing and analyzed using the Genescloud platform^1^ accessed on June 20, 2024 (Guo et al., 2022) to assess the microbial community in the V3-V4 region. Initially, nucleic acids were extracted using the TIANamp Stool DNA Kit (D5625-01) (Omega Bio-Tek, Norcross, GA, United States), followed by agarose gel electrophoresis to determine the molecular size of the nucleic acids. The DNA was then quantified using a spectrophotometer (ND-2000; NanoDrop Technologies, Wilmington, DE, United States). Primers 338F (5′-CCTAYGGGRBGCASCAG-3′) and 806R (5′-GGACTACNNGGGGTATCTAAT-3′) were employed to target the V3-V4 variable region of the bacterial 16S rRNA gene (Fischer et al., 2016). PCR amplification of the 16S rRNA gene was conducted according to the protocol described by Magoč and Salzberg (2011). Fragmentation was performed using a Covaris ME220 ultrasonic crusher, followed by PCR (Dang et al., 2022). Total DNA was subjected to full-length 16S rRNA gene sequencing on a PacBio Sequel II platform (Shanghai Lingen Biotechnology Co., Ltd.) using the Sequel II System Application Software v10.0. Raw reads were processed with the DADA2 pipeline (Callahan et al., 2016) as implemented in QIIME 2 v2022.2. Briefly, CCS (circular-consensus) reads were demultiplexed, quality-filtered (Q-score ≥ 20), and dereplicated; error rates were learned and denoised to yield amplicon sequence variants (ASVs). Chimeras were removed with the consensus method. ASVs were mapped against the Greengenes 13_8 99% reference (Agnihotry et al., 2020) for initial taxonomic placement, followed by assignment with the RDP classifier v2.13 (Cole et al., 2014) trained on the same database, providing annotations from phylum to genus. Alpha-diversity metrics [Chao1, ACE, Shannon, Simpson, and Observed (ASVs) were calculated in QIIME 2 after rarefaction to 8,000 reads per sample].

#### 2.1.3 Sensory evaluation

After opening the silo, we conducted a sensory evaluation using the traditional DLG (German Agricultural Society) silage scoring standard (Hao et al., 2024), focusing on Odor, Texture and Color. The detailed criteria are given in Table 1. On-site sensory evaluation was carried out by a panel of four to five trained assessors. The overall quality of silage fermentation was then determined from these scores. The maximum scores for Odor, Texture, and Color are 14 points, 4 points, and 2 points, respectively. Samples were presented in random order and coded so that panelists were blind to treatment identity. Consensus scores were averaged for statistical analysis.

**Table 1:** Standards for silage sensory evaluation.

Items	Marking scheme			Scores
Odor indicator	There is no odor of butyric acid, and the flavor is characterized by aromatic fruitiness rather than a pronounced bread taste			14
	A faint odor of butyric acid, Faint butyric-acid odor with mild sourness and subtle aroma			10
	A strong odor of butyric acid, characterized by a pungent, burnt, or musty scent			4
	Strong butyric-acid or ammonia odor; acidic taste almost absent			2
Textural indicator	The stem-and-leaf structure is well maintained			4
	Poorly maintained stem-and-leaf structure			2
	The stem-and-leaf structure is very poorly maintained, and there is mild mold contamination			1
	Stem and leaf rot or severe contamination			0
Color indicator	Similar to raw materials, it appears light brown after drying			2
	A slightly discolored light yellow or brownish hue			1
	Severe discoloration, characterized by dark green or brownish-yellow hues, accompanied by a strong, moldy odor			0
Total score	16–12	10–15	5–9	0–4
Hierarchy	Grade 1: Excellent	Grade 2: Fair	Grade 3: Moderate	Grade 4: Spoiled

### 2.2 Animals feeding treatments

The trial commenced on December 17, 2023, in the Dazi District of Lhasa, Xizang. Twenty healthy Xizang Holstein cows were selected from multiple farms. These cows had an average milk production of 3.67 ± 1.96 liters, an average parity of 2.00 ± 0.60, and an average of 260.00 ± 6.86 days in milk. The cows were randomly assigned to blocks based on days in milk and subsequently allocated to either a control group (*n* = 10) or a treatment group (*n* = 10). Throughout the study period, all dairy cows were housed in individual pens at their respective farmers’ residences. Both groups received the same concentrate feed. The composition and nutrient levels of the concentrate are shown in Table 2. The feed was prepared by manually mixing the concentrate with the roughage before feeding. Based on our preliminary trials of whole-plant maize silage fermentation, it was found that extending fermentation duration improves feed quality characteristics, such as increased crude protein and lactic acid content. Consequently, Silage fermented for 60 days was used as feed for dairy cows. The control group received maize silage that had been ensiled for 60 days without additives, whereas the treatment group received maize silage ensiled for 60 days with microbial additives. Each dairy cow was offered 10 kilograms of maize silage and 2.5 kilograms of concentrate daily on a dry-matter basis, achieving a concentrate-to-roughage ratio of 2:8. The trial included an 8-day adaptation period followed by a 25-day formal feeding period. During the final 3 days of the experiment, milk production was recorded using the PUCHUN JS100-1 high-precision electronic scale. Measurements were taken at two specific time intervals: from 7 to 8 a.m. and from 3 to 4 p.m. each day. The collected milk samples were subsequently analyzed for their components. Indicators as milk fat, milk protein, lactose, non-fat solids, urea nitrogen, and total dry matter were determined using the milk composition analyzer (MilkoScan FT + 200, Type 76150).

**Table 2:** Dairy cow concentrate formulation and nutritional levels (dry matter basis) %.

Items	Content
Ingredients	
Corn	13.16
Soybean meal	50.00
Cottonseed meal	16.97
Fat powder	3.76
CaCO_3_	1.38
CaHPO_4_	5.73
Premix^1^	5.00
Salt	4.00
Nutrient level (%)^2^	
CP	31.40
Ca	2.42
P	1.84
NDF	11.94
ADF	6.65

1 One kg of premix contained the following: VA 3,000 IU, VD 1,200 IU, VE 50 mg, Se 0.5 mg, Mn 160 mg, Cu 260 mg, Fe 3,200 mg.

2 Concentrate Feed Nutrient levels was all measured values. CP, crude protein; Ca, Calcium; p, Phosphorus; NDF, neutral detergent fiber; ADF, acid detergent fiber.

### 2.3 *In vitro* rumen fermentation treatment

#### 2.3.1 Treatment animals

The silage maize samples collected at different time points were subjected to a 48-h *in vitro* fermentation treatment. Rumen fluid was collected from three healthy Xiangxi yellow cattle with permanent fistulae. On a total-mixture dry-matter basis (100% DM), the concentrate portion supplied: Each animal was fed daily with concentrate supplements consisting of 10% maize, 3.48% soybean meal, 11.2% rice, 5.6% wheat bran, 2.8% oil bran, 0.8% sprayed maize hulls, 1.2% soybean germ flour, 1.2% Brown rice, 0.8% Soybean husk, 0.12% Rumen bypass fat powder, 0.32% expanded urea, 0.5% triticale and 1.98% premix, along with 60% whole silage maize. The fermentation substrates included maize silage samples from 0, 30, and 60 days. The fistulated cattle were fed at 08:00 and 18:00 each day and had ad libitum access to fresh water. The barn was maintained under hygienic and epizootic standards; ambient temperature ranged from 6 to 26 °C, lighting was natural, and routine deworming and husbandry practices were applied.

#### 2.3.2 *In vitro* rumen fermentation

On the day of the trial, fresh rumen fluid was collected from the fistulated cows 1 h before morning feeding. The fluid was transferred to a thermos flask pre-flushed with CO_2_ and immediately filtered through six layers of sterile cheesecloth, yielding 600 mL of filtrate. Following Menke et al. (1979), a 2,400 mL buffer solution was prepared by adding distilled water and the appropriate buffer constituents to a continuous-flow, 39.5 °C water bath. The filtered rumen fluid, reducing agent and trace elements were then added sequentially until the mixture became colorless. The combined solution was gently mixed under a continuous CO_2_ stream to produce 3 L of anaerobic *in vitro* rumen fluid. For each treatment, 0.6 g of fermentation substrate was weighed into 150 mL serum bottles, and 60 mL of the freshly prepared rumen fluid was added. Each treatment comprised four replicate bottles cultured under strictly anaerobic conditions at 39.5 °C. Fermentation was conducted using a fully automated *in vitro* rumen simulation apparatus (Wang et al., 2016). The degassing pressure was set at 10.0 kPa, with released gasses automatically entering a gas chromatograph (Agilent 7890A, Agilent Technologies, Palo Alto, United States) for measurement of total gas production concentration.

#### 2.3.3 Collection and measurement of rumen fluid

After fermentation was completed at 48 h, the fermentation broth was sequentially collected from the fermentation flasks, and the pH was quickly measured using a PHS-3C pH meter (Mettler-Toledo Instruments). A 2 mL aliquot of the shaken fermentation broth was centrifuged at 4 °C and 15,000 rpm for 10 min. Subsequently, 1.5 mL of the supernatant was transferred, and 0.15 mL of 25% metaphosphoric acid was added to it. This mixture was thoroughly mixed and stored at −20 °C. The supernatant was thawed at 4 °C after being removed from the −80 °C freezer. The thawed supernatant was then centrifuged again to determine the concentrations of ammonia (NH_3_-N) and volatile fatty acids (VFAs) in the rumen fluid. The residue of the fermentation broth, after being filtered through gauze, was placed in an aluminum container and dried at 105 °C until a constant weight was achieved. The dry matter degradation (DMD) and total gas production (TGP) were calculated based on the weight of the fermentation substrate before fermentation and the weight of the residue after the drying process, following the method described by Zhang et al. (2018). The gasses produced from *in vitro* fermentation were analyzed using a gas chromatograph (Agilent 7890 A, Agilent Inc., Palo Alto, California, United States). The gas content generated from the fermentation of the substrate was calculated using NLREG software and analyzed by fitting the model provided by Wang et al. (2013a, 2016).

#### 2.3.4 Data analysis

The statistical analyses were conducted utilizing SPSS v.20.0 (SPSS Inc., Chicago, United States). A two-factor analysis of variance (ANOVA) was conducted on the two factors of days and treatment using a general linear model (GLM). Comparative analyses between experimental groups were performed using one-way ANOVA. The data of bacterial compositions were analyzed by nonparametric test models, and the differences were judged by the Kruskal–Wallis test. Results are presented as means with standard error of the mean (SEM). *p* ≤ 0.05 indicates that a finding was significant, and 0.05 < *p* ≤ 0.10 indicates a tendency to be significant.

## 3 Results

### 3.1 Sensory evaluation and silage quality of whole-plant maize

As illustrated in Table 3, the ratings for odor, texture, color, and overall score of the treatment groups were all classified as excellent. Furthermore, the odor and overall score ratings of the treatment groups were significantly higher 6.25 and 7.46% (*p* < 0.05) at 60 days compared to those of the control group. There was no interaction effect on the sensory evaluation of silage maize in terms of time and days (*p* > 0.05). As can be seen from Table 4, at the 30th and 60th d of fermentation, the CP content of the treatment group was significantly increased 31.21 and 28.86% (*p* < 0.01) compared with the control group, and there was no significant difference (*p* > 0.05) in DM, EE, Ash, NDF, and ADF between the two groups. The interaction between the duration of fermentation and the applied treatments significantly influenced the CP content (*p* < 0.05). As can be seen from Table 5, the pH value of the treatment group decreased significantly 31.95 and 7.59% (*p* < 0.01) at the days 30 and 60 of silage maize fermentation. As fermentation time increased, by days 30 and 60, the LA content and AA content in the treatment group became significantly higher than that in the control group (*p* < 0.01). The interaction between the days of fermentation and the treatment had significant effects (*p* < 0.05) on pH, LA, and AA. The pH value, NH_3_-N, LA, and AA showed significant differences across various fermentation days (*p* < 0.01). Additionally, the concentrations of NH_3_-N, LA, and AA were significantly higher at days 30 and 60 compared to those at day 0.

**Table 3:** The sensory evaluation scores of whole-plant maize silage after microbial additive treatment.

Items	30 day		60 day		SEM^1^	*p*-value^2^		
	Con	Treat	Con	Treat		Day	Treat	D × T
Odor indicator	12.90	13.30	12.80^b^	13.60^a^	0.26	0.623	0.043	0.522
Textural indicator	2.80	3.10	3.00	3.20	0.22	0.501	0.268	0.821
Color indicator	1.66	1.80	1.56	1.92	0.12	0.934	0.052	0.368
Total score	17.36	18.20	17.42^b^	18.72^a^	0.39	0.472	0.015	0.567
Hierarchy	Grade 1 Excellent	Grade 1 Excellent	Grade 1 Excellent	Grade 1 Excellent	–	–	–	–

1 SEM, standard error of mean.

2 *p* ≤ 0.05 is considered statistically significant.

^a,b^ Means within a row with different superscripts differ significantly (*P* < 0.05).

**Table 4:** The impact of microbial additives on the nutritional composition of whole-plant maize silage.

Items^1^	0 day		30 day		60 day		SEM^2^	*p*-value^3^		
	Con	Treat	Con	Treat	Con	Treat		Day	Treat	D × T
DM (%)	22.71	22.52	22.87	24.45	22.77	24.17	0.84	0.423	0.182	0.514
CP (%/DM)	6.35	6.66	6.60^b^	8.66^a^	7.31^b^	9.42^a^	0.27	<0.001	<0.001	0.003
EE (%DM)	5.33	5.08	5.33	5.82	5.16	6.00	0.38	0.529	0.247	0.352
Ash (%/DM)	4.76	5.04	4.65	4.74	4.92	5.03	0.35	0.710	0.577	0.954
NDF (%/DM)	38.61	37.87	36.25	36.80	37.88	37.44	1.40	0.465	0.855	0.890
ADF (%/DM)	25.00	24.74	24.50	25.93	24.65	24.01	1.54	0.846	0.888	0.778

1 DM, Dry Matter; CP, Crude Protein; EE, Crude Fat; DNF, Neutral Detergent Fiber; ADF, Acid Detergent Fiber.

2 SEM, standard error of mean.

3 *p* ≤ 0.05 is considered statistically significant.

^a,b^ Means within a row with different superscripts differ significantly (*P* < 0.05).

**Table 5:** Effect of microbial additives on the fermentation quality of whole-plant maize silage.

Items^1^	0 day		30 day		60 day		SEM^2^	*p*-value^3^		
	Con	Treat	Con	Treat	Con	Treat		Day	Treat	D × T
pH value	5.87	5.77	5.79^a^	3.94^b^	3.96^a^	3.56^b^	0.12	<0.001	<0.001	<0.001
NH_3_-N (mg/kg)	0.09	0.07	0.29	0.22	0.17	0.24	0.02	<0.001	0.938	0.019
LA (mg/mL)	17.78^a^	10.92^b^	11.24^b^	75.09^a^	31.39^b^	62.27^a^	0.59	<0.001	<0.001	<0.001
AA (mg/mL)	0.19	0.20	3.27^b^	4.09^a^	2.10^b^	4.98^a^	0.03	<0.001	<0.001	<0.001

1 NH_3_-N, Ammonia-Nitrogen; LA, Lactic acid; AA, Acetic acid.

2 SEM, standard error of mean.

3 *p* ≤ 0.05 is considered statistically significant.

^a,b^ Means within a row with different superscripts differ significantly (*P* < 0.05).

### 3.2 Effect of microbial additives on bacterial diversity of whole-plant maize silage

**Figure 1:**
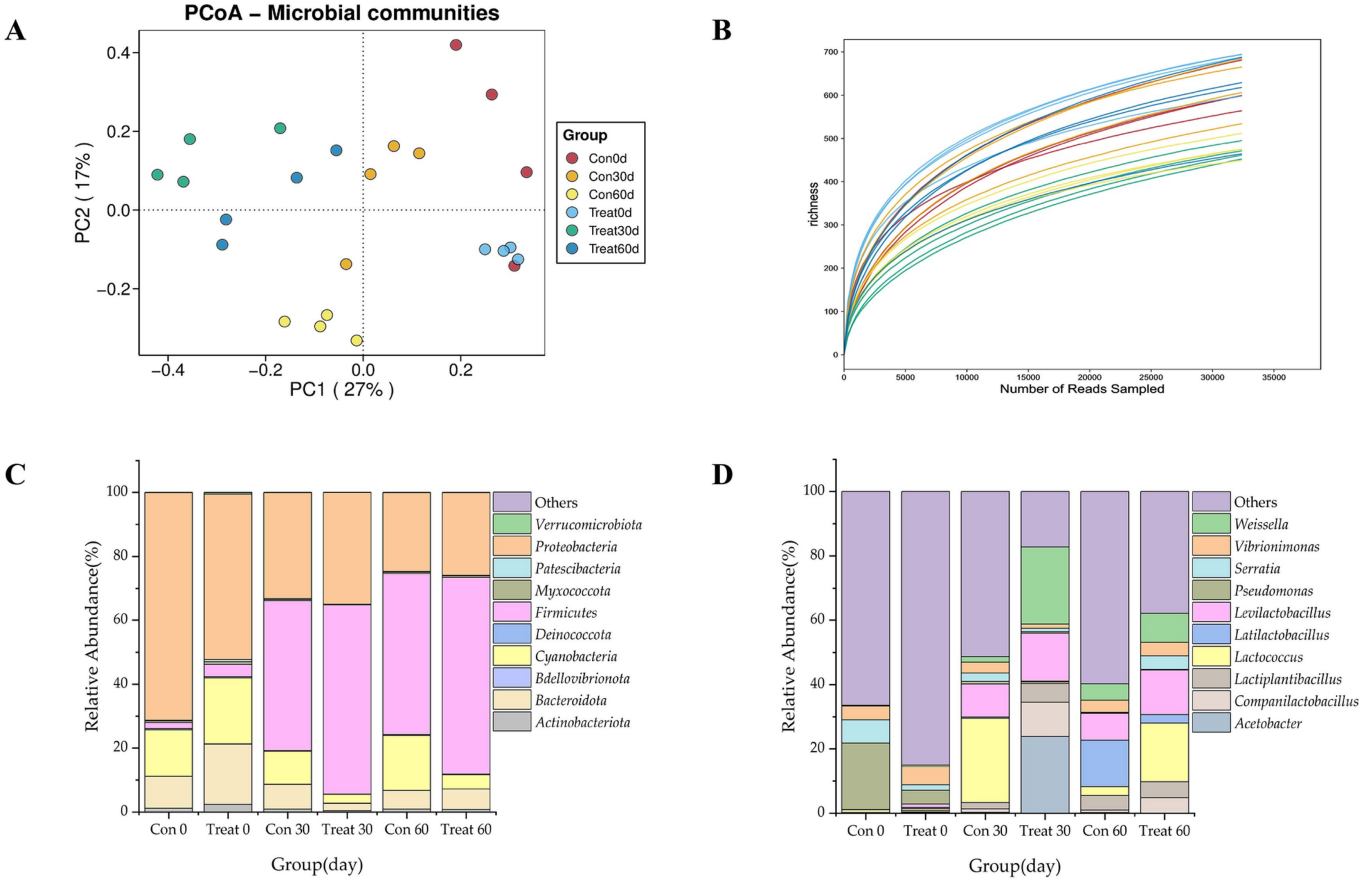
**(A)** Results of principal coordinate analysis (PCoA) of OTU levels at different time points in silage maize by microbial additives; **(B)** rarefaction curves based on OTUs, each curve represents one sample; **(C)** stacked plot analysis of differences in bacterial phylum-level flora of whole maize after fermentation with microbial additives; **(D)** stacked plot analysis of differences in bacterial genus-level flora of whole maize after fermentation with microbial additives.

Observed species, Chao1, ACE and Faith’s PD were significantly lower 24.39, 21.32, 17.76, and 17.55% (*p* < 0.05) in the treatment group at 30 days of fermentation. In addition to Simpson, the indices of Observed species, Chao1, ACE, Shannon, and Pielou_J were all found to interact significantly with silage fermentation days and treatment conditions (*p* < 0.05). There was no statistically significant difference in Faith’s PD between the two groups on the 60th day of fermentation (*p* > 0.05). The ACE index was significantly higher at 0 and 30 days of fermentation compared to 60 days (*p* < 0.05) (Table 6). As illustrated in Figure 1A, the samples from various subgroups at each time point showed staggered sample distribution with no clear clustering. Meanwhile, the results of the principal coordinate analysis (PCoA) conducted at the operational taxonomic unit (OTU) level using the Bray-Curtis algorithm indicate that the first axis of interpretation (PCo1) accounted for 27% of the variance, while the second axis (PCo2) accounted for 17%. These results suggested that the nutrient composition of silage varied among different subgroups at each time point, and there were also minor differences in the microbial composition of the silage. As illustrated in Figure 1B, each sample contained more than 30,000 reads, and the species richness had largely reached a plateau, suggesting that the vast majority of species present in each sample were detected, thereby indicating a high level of data reliability. As illustrated in Figure 1C, at the Phylum level, the phyla Bdellovibrionota, Firmicutes, Patescibacteria, and Verrucomicrobiota exhibited significantly higher abundances (*p* < 0.05) in the treatment group on the 0th day of fermentation. Conversely, on the 30th day of fermentation, the phyla Actinobacteriota, Bacteroidota, Deinococcota, Myxococcota, and Patescibacteria demonstrated significantly lower abundances (*p* < 0.05). By the 60th day of fermentation, the Firmicutes phylum showed a significant increase 27.92% (*p* < 0.05), while a trend toward an increase in Patescibacteria was observed (*p* = 0.050). As illustrated in Figure 1D, at the bacterial genus level, on the 0th day of fermentation, the relative abundances of *Companilactobacillus*, *Latilactobacillus*, *Levilactobacillus*, and *Weissella* in the treatment group were significantly higher than those in the control group (*p* < 0.05). Meanwhile, the levels of *Lactococcus*, *Vibrionimonas*, and other unclassified genera showed a significant decrease (*p* < 0.05). On the 60th day of fermentation, the genera *Acetobacte*r, *Latilactobacillus*, and other unclassified genera showed a significant reduction in abundance 95.23 and 81.96% (*p* < 0.05), while the genus *Weissella* showed a significant increase 78.19% (*p* < 0.05).

**Table 6:** The impact of microbial additives on the diversity of bacterial communities in whole-plant maize silage.

Items	0 day		30 day		60 day		SEM^1^	*p*-value^2^		
	Con	Treat	Con	Treat	Con	Treat		Day	Treat	D × T
Observed species	607.00	667.00	620.25^a^	469.00^b^	476.75	598.00	28.21	0.004	0.669	<0.001
Chao1	773.16	805.63	818.65^a^	644.10^b^	621.46	764.35	35.18	0.042	0.993	0.001
ACE	781.88	822.08	812.63^a^	668.31^b^	629.49	770.35	32.62	0.019	0.651	0.001
Shannon	3.78	4.33	3.74	2.92	3.50	3.87	0.22	0.015	0.855	0.013
Simpson	0.93	0.93	0.92	0.85	0.91	0.94	0.03	0.194	0.616	0.227
Pielou_J	0.59	0.67	0.58	0.47	0.57	0.61	0.03	0.027	0.927	0.035
Faith’s PD	29.62^b^	35.33^a^	30.89^a^	25.47^b^	27.53	31.11	1.39	0.017	0.269	0.002

1 SEM, standard error of mean.

2 *p* ≤ 0.05 is considered statistically significant.

^a,b^ Means within a row with different superscripts differ significantly (*P* < 0.05).

### 3.3 Effect of feeding Holstein cows on milk production and milk composition

As can be seen from Table 7, there was a trend toward higher milk fat percentage (*p* = 0.055; Table 7), significant increase in milk protein percentage and total solids percentage content 14.06 and 8.30% (*p* < 0.05), and no significant differences were observed in the milk production, somatic cells, solid not fat percentage, and urea nitrogen in the treatment group compared to the control group (*p* > 0.05).

**Table 7:** Effect of feeding microbial additives whole-plant maize silage on milk production and milk composition of Holstein cows in Xizang.

Items	Group		SEM^1^	*p*-value^2^
	Con	Treat		
Milk production (kg/d)	3.92	4.40	0.98	0.627
Somatic cell count (10^3^/mL)	389.83	178.57	157.88	0.208
Milk fat percentage (%)	4.20	4.97	0.35	0.055
Milk protein percentage (%)	3.13^b^	3.57^a^	0.18	0.031
Milk lactose percentage (%)	4.65	4.54	0.09	0.280
Solid Not Fat (%)	8.80	9.12	0.18	0.108
Urea nitrogen (mg/dL)	11.40	9.79	1.51	0.307
Total Solid (%)	13.01^b^	14.09^a^	0.47	0.040

1 SEM, standard error of mean.

2 *p* ≤ 0.05 is considered statistically significant.

^a,b^ Means within a row with different superscripts differ significantly (*P* < 0.05).

### 3.4 Effect of substrate degradation, gas production and volatile fatty acids in rumen fermentation *in vitro*

As can be seen from Table 8, on the 0th d of fermentation, compared with the control group, the contents of MCP, TVFA, butyrate and the A: P ratio were significantly higher (*p* < 0.05), and the contents of isobutyrate and isovalerate were significantly lower (*p* < 0.05) in the treatment group with silage addition; on the 30th d of fermentation, compared with the control group, the contents of rumen pH, TVFA, propionate, isobutyrate, butyrate, isovalerate, and valerate was significantly lower (*p* < 0.05) but the contents of acetate and TGP was significantly increased (*p* < 0.05); on the 60th d of fermentation, DMD, NH_3_-N, TVFA, butyrate and valerate was significantly higher 9.92, 22.24, 2.54, 15.72, and 14.66% (*p* < 0.05) and acetate content was significantly lower (*p* < 0.05). There was no interaction effect among TGP, pH value, MCP NH_3_-N, isobutyrate and isovalerate (*p* > 0.05).

**Table 8:** Effect of microbial additives on the *in vitro* fermentation characteristics of whole-plant maize silage.

Items^1^	0 day		30 day		60 day		SEM^2^	*p*-value^3^		
	Con	Treat	Con	Treat	Con	Treat		Day	Treat	D × T
DMD (%)	70.68	71.67	70.45^b^	72.52^a^	67.65^b^	74.36^a^	0.66	0.857	<0.001	0.002
TGP (mL/g DM)	350.82	347.16	345.90^b^	387.14^a^	332.49	364.05	9.52	0.123	0.008	0.071
pH value	6.25	6.23	6.25^a^	6.20^b^	6.24	6.19	0.02	0.534	0.019	0.722
MCP (mg/mL)	0.40^b^	0.48^a^	0.48^b^	0.51^a^	0.53	0.54	0.02	<0.001	0.018	0.153
NH_3_-N/ (mg/kg)	10.02	10.45	12.98	13.46	14.39^b^	17.59^a^	0.87	<0.001	0.068	0.24
TVFA (mmol/L)	114.11^b^	117.20^a^	122.84^a^	119.45^b^	119.07^b^	122.10^a^	0.62	<0.001	0.089	<0.001
Acetate (%)	66.91	66.97	61.83^b^	63.73^a^	63.32^a^	61.64^b^	0.14	<0.001	0.396	<0.001
Propionate (%)	21.65	21.48	25.06^a^	23.5^b^	24.21	24.35	0.13	<0.001	<0.001	<0.001
Isobutyrate (%)	0.90^a^	0.83^b^	0.99^a^	0.95^b^	0.94	0.93	0.01	<0.001	0.001	0.077
Butyrate (%)	8.00^b^	8.24^a^	8.98^a^	8.87^b^	8.59^b^	9.94^a^	0.09	<0.001	<0.001	<0.001
Isovalerate (%)	1.40^a^	1.31^b^	1.62^a^	1.49^b^	1.51	1.49	0.03	<0.001	0.001	0.112
Valerate (%)	1.15	1.17	1.53	1.38	1.43^b^	1.64^a^	0.02	<0.001	0.147	<0.001
A: P	3.09	3.12	2.47^b^	2.70^a^	2.62^a^	2.53^b^	0.02	<0.001	0.001	<0.001

1 DMD, Dry Matter Degradation rate; TGP, Total Gas Production; MCP, Microbial Crude Protein; NH_3_-N, Ammonia-Nitrogen; TVFA, Total Volatile Fatty Acids; A: P, Acetate-to-Propionate ratio.

2 SEM, standard error of mean.

3 *p* ≤ 0.05 is considered statistically significant.

^a,b^ Means within a row with different superscripts differ significantly (*P* < 0.05).

Significant interactions among TVFA, acetate, propionate, butyrate, and valerate were observed between the two groups (*p* < 0.01).

## 4 Discussion

### 4.1 Effect of fermentation of whole-plant maize with silage addition on silage quality

Silage sensory evaluation is employed to assess the quality of silage by examining its odor, texture, and color, thereby facilitating a preliminary judgment regarding its condition. In the present study, as fermentation time was extended, the sensory evaluation of both the control group and the treatment group initially yielded excellent results. This finding aligns with the research conducted by Jaeger et al. (2024), which indicates that certain amounts of LAB and soluble sugars are naturally present in whole maize raw material. These LAB can rapidly proliferate under suitable anaerobic conditions, utilizing the sugars to perform lactic acid fermentation, thereby ensuring the quality of the silage. Although DM content, WSC, epiphytic microbes were not determined in this study, reference values collected from the same plateau area, cultivar and harvest period are available. Wu et al. (2025) reported 28% DM, the WSC is 8.7%, and the lactic acid bacteria are 3 log_10_ CFU/ g^−1^ FM. Consequently, it is reasonable to assume that the substrate in the current trial contained sufficient fermentable carbohydrates and a natural LAB population adequate for successful ensiling.

The essence of silage is that feedstuffs decompose sugars through LAB fermentation to produce lactic acid and some of acetic acid to make pH lower to inhibit microbial activities to achieve the purpose of long-term preservation of green fodder, and its nutritional composition and fermentation quality directly reflect the quality of silage, and the fermentation quality of the LA, AA and PA content is the key index of the fermentation of silage (Wang et al., 2025). Higher crude protein (CP) content in silage generally indicates better nutritional value and improves animal production performance. In this experiment, the CP contents in the two treatment groups were significantly higher than that of the control group after 30 and 60 days of fermentation. This result showed consistency with previous studies, and the possible reason for this phenomenon might be that high-quality lactic acid bacteria rapidly produced acid to inhibit proteolytic bacteria, while the growth of *Saccharomyces cerevisiae* itself contributed microbial protein, ultimately resulting in a net increase in crude protein in the treated group (Liu Y. et al., 2025; Wang et al., 2024; Yu et al., 2023). The observed increase in protein levels can be attributed to the ability of the *Saccharomyces cerevisiae* and LAB microbial additives to further lower the pH of the silage environment and to produce a significant quantity of LA during the later stages of fermentation. Notably, the LA content in the experimental group reached its peak on the 30th day, while in terms of fermentation days, which is consistent with the findings of Khota et al. (2017). This further explains the observed increase in lactic acid content and the corresponding decrease in pH value in the silage fermentation process of this experiment (Ma et al., 2024). Both indicators reflect the enhanced fermentation quality of the silage treatment (Li et al., 2022; Xia et al., 2023). This enhancement not only aids in the preservation of nutrients such as DM and CP but also inhibits the growth of detrimental microorganisms. The AA content of the treatment group increased significantly at both 30 days and 60 days of fermentation. On the three different fermentation days, the AA content on day 0 was significantly lower than that on days 30 and 60. Furthermore, compounds such as AA, PA, sodium benzoate, and potassium sorbate, which serve as chemical preservatives, can effectively control mold contamination in food and feed, thereby improving the overall quality of maize silage fermentation (Nair et al., 2019). In this study, the concentration of acetic acid increased with prolonged fermentation time, aligning with the findings of Elferink et al. (1999). Notably, butyric acid (BA) was not detected in the entire silage material, suggesting that the silage processing adhered to standardized protocols. Additionally, LA was found to inhibit the secretion of BA by *Clostridium butyricum* (Xu et al., 2017).

### 4.2 Effect of silage fermentation on bacterial microorganisms

The declines of Observed species, Chao1, and ACEin the treated group at 30 days of fermentation in this study indicated that the exogenous addition of the compound bacterial agent significantly reduced the richness of bacterial species. This indicates that the addition of exogenous compound bacterial preparations significantly reduces the richness of bacterial species, but it does not imply a weakening of community function or stability. The goal of silage fermentation is to rapidly establish an acidic environment and inhibit aerobic spoilage bacteria such as actinomycetes and cyanobacteria (Liu B. et al., 2019; Wang et al., 2024) found in their study on barley silage that Chao1 and ACE were significantly reduced after treatment with Lactobacillus, which was attributed to the screening effect of low pH value on non-target bacteria. This mechanism can not only eliminate redundant species but also maintain community stability, laying a foundation for the improvement of fermentation quality in the subsequent 60 days. Usually, Shannon index and the Simpson index show a negative correlation trend in most ecosystems (Yan et al., 2023). However, no significant changes were observed in the Simpson and Shannon indices between two groups in the current study. This discrepancy might be due to the synergistic effect of *Saccharomyces cerevisiae* and lactic acid bacteria. Meanwhile, this observation aligns with the findings of Wang et al. (2024), who also reported a lack of an inverse relationship between these indices in maize treated with lactic acid bacteria. In addition, the observed species, ACE, Shannon, Pielou_J, and Faith_Pd decreased significantly with the extension of fermentation days. This reduction in richness was indicative of a decrease in the abundance of microbial populations and communities, which can be attributed to the gradual predominance of acid-resistant bacteria throughout the fermentation process (He et al., 2019). Faith’s Pd surged on day 0—when live *Saccharomyces cerevisiae* and LAB were introduced—indicating a burst of phylogenetically divergent, ecologically distinct taxa, but declined by day 30 as fermentation selected for functionally similar lineages, converging the tree and reduced ecofunctional diversity (Armstrong et al., 2021).

Silage fermentation is driven by bacteria: Bdellovibrionota and Firmicutes degrade fiber under anoxia, accelerating plant-cell-wall breakdown and suppressing pathogens such as *E. coli* and Salmonella (Dong et al., 2022; Ni et al., 2018; Wang et al., 2021). The increased abundance of Firmicutes observed at both 0 days and 60 days of fermentation suggests that the normal activity of rumen fiber-degrading bacteria contributes to nutrient provision and enhances the population of fiber-degrading bacteria, promotes the growth of rumen microorganisms, and enhances nitrogen retention (Herremans et al., 2019). Moreover, the increase in NH_3_-N levels observed in the rumen *in vitro* fermentation of the ensiled maize substrate after 60 days of fermentation in this study corroborates this phenomenon. Additionally, members of Deinococcota and Myxococcota face competitive disadvantages against LAB, while nutrients, including soluble carbohydrates, are gradually depleted, leading to a reduction in available nutrients (Kurashita et al., 2024; Liu Z. D. et al., 2019; Zhang et al., 2023). Patescibacteria, enriched in hypoxic aquifers, rapidly convert plant polysaccharides to mannose, starch and cellulose. These products peaked on day 0, then fell by days 30 and 60, mirroring the supply of fermentable substrate in the plateau valley. Microbial inoculants accelerated the flux: sugars fuelled LAB acidogenesis, lactate accumulated, pH dropped and Patescibacteria lost competitiveness, so their abundance declined (Dong et al., 2021; Herrmann et al., 2019; Hosokawa et al., 2021; Jonas et al., 2025). Verrucomicrobiota, commonly found in soil, aquatic environments, and animal intestines, contribute to intestinal health by breaking down polysaccharides and producing short-chain fatty acids such as butyric acid (Tan et al., 2025). This aligns with the present experiment, where using 0-day fermented maize silage as substrate led to elevated butyric acid levels during *in vitro* rumen fermentation. The microbiota present on the surface of raw materials during silage fermentation primarily consists of *Acetobacter* spp., *Clostridium* spp., spoilage fungi, molds, and yeasts (Zhang et al., 2025). On day 0 of fermentation, the addition of microbial additives in the treatment group facilitated the rapid colonization of ecological niches by functional LAB, including *Companilactobacillus*, *Lactiplantibacillus*, *Latilactobacillus*, and *Levilactobacillus*. This suggests that acidic silage enhances the permeability of the outer membrane of Gram-positive bacteria, thereby altering the microbial community structure (Kahraman-Ilıkkan, 2024). Previous studies have also demonstrated that shortly after inoculation with microbial additives, the abundance of LAB increases significantly, which can effectively suppress the indigenous microbial flora (Su et al., 2024). During the silage process, these heterofermentative bacteria, such as *Lactiplantibacillus*, *Latilactobacillus*, and *Levilactobacillus*, produce volatile fatty acids that contribute to long-term preservation (Su et al., 2024). By day 30 of fermentation, acid-tolerant genera such as *Lactococcus* and *Vibrionimonas* exhibited a significant decline. This may be attributed to the substantial acid production in the experimental group within the first 30 days, leading to a pH drop that induced acid stress and consequently reduced the abundance of these genera (QU et al., 2023; Xin et al., 2023). *Acetobacter*, commonly found in fermented grains, spoiled fruits, vinegar, and alcoholic beverages, possesses a strong oxidizing capability that can lead to the spoilage of feedstuffs. It is also capable of oxidizing ethanol to produce acetic acid and further oxidizing acetic acid to generate carbon dioxide (Gomes et al., 2018; Guan et al., 2020). Notably, the abundance of *Acetobacter* was significantly reduced by the 60th day of fermentation, suggesting that the addition of silage may mitigate spoilage in whole-plant maize silage (Han et al., 2024). *Weissella,* a Gram-positive bacterium, a facultative anaerobic rod-shaped bacterium, commonly found in high-acid or fermented environments such as grains and pickles. Its metabolic product is mainly lactate. Metabolizes carbohydrates through the hexose-monophosphate and phosphotransacetylase pathways. Its primary end products include lactic acid, carbon dioxide, acetic acid, and ethanol (Liu C. et al., 2025). The incorporation of microbial additives in this experiment accelerated the decrease in pH of whole-plant maize silage and shifted the fermentation pattern from lactic acid to acetic acid fermentation, a change that may be linked to the increased abundance of *Weissella* (Fusco et al., 2023).

### 4.3 Effect of feeding Holstein cows on milk production and milk composition

*Saccharomyces cerevisiae* and LAB are beneficial microorganisms that inhibit harmful silage microorganisms and are utilized as direct feed for dairy cows. These organisms are also commonly employed in the production of fermented foods and feeds due to their capacity to modify the microbial flora and enhance the flavor of the food. In this study, there was no significant change in milk production between the two groups of dairy cows. This result is inconsistent with the previous report by Garnsworthy et al. (2025) that the milk production in the high-yielding dairy cows treatment group increased by 5.47% after adding *Saccharomyces cerevisiae* compared with the control group. This discrepancy might be attributed to the difference in the breeding environment and physiological stage of dairy cows. Because the fact that the experimental dairy cows in this study were raised in a high-altitude, low-temperature and low-oxygen area and were in the late lactation stage (~260 d), the response of milk production might have been limited. In the current experiment, cows in the microbial additives group demonstrated a significant increase in their milk protein percentage, indicating an improvement in milk composition. Additionally, there was a trend toward higher milk fat percentage, which aligned with the findings of Poppy et al. (2012). The development and scientific utilization of silage maize resources at high altitudes are advantageous for enhancing the growth performance of highland grass-fed livestock, enriching the feed resources available for highland livestock husbandry, and improving the economic benefits for farmers and herdsmen. Furthermore, this approach facilitates the effective utilization of high-altitude silage maize (Li et al., 2024). Research has demonstrated that ensiled maize can be fed to dairy cows and other herbivorous livestock following fermentation, thereby ensuring normal growth and enhancing production performance (Beltrán et al., 2019; Waziri and Uliwa, 2020). Consequently, this practice can effectively alleviate the pressure on plateau grasslands and mitigate the occurrence of weight loss in livestock.

### 4.4 *In vitro* fermentation of volatile fatty acids, substrate degradation and gas production

The rumen constitutes a critical component of ruminant metabolism. The stability of the rumen’s internal environment is influenced by various factors, including pH, NH_3_-N and VFA concentrations in the rumen (Dijkstra et al., 2012; Li et al., 2021). In the present study, the *in vitro* rumen pH was sustained within the normal range of 5.5 to 7.5 (Wanapat et al., 2014). However, it was observed that the rumen pH in the treatment group was significantly lower than that of the control group after 30 days of fermentation using microbial additives. This reduction may be attributed to an increase in lactic acid content within the fermentation substrate. Microbial crude protein (MCP) in the rumen plays a vital role in the synthesis and catabolism of proteins in the animal (Anggraeny et al., 2021). The concentration of NH_3_-N serves as a direct indicator of the nitrogen metabolism activity level within the organism to some extent (McDonald and Edwards, 1976). The significant increase in rumen MCP content observed in the treatment group at both 0 and 30 days of fermentation, with no significant change at 60 days. However, in terms of the change in fermentation days, the average value of MCP reached its peak at 60 days, suggests that rumen microorganisms can accelerate the synthesis of microbial proteins by utilizing substrates such as NH_3_-N and amino acids. This process improves the efficiency of nitrogen utilization in the rumen, thereby enhancing the capacity for MCP synthesis (Sok et al., 2017). Furthermore, the concentration of NH_3_-N in the experimental group exhibited an increasing trend by the 60th day. However, the NH_3_-N levels were notably higher on days 30 and 60 than day 0 across different fermentation days. This increase may be attributed to the gradual accumulation of amino acids, nitrogenous salts (including nitrates and nitrites), purines, and pyrimidines in the plant material, as well as the mortality of microorganisms present in the fermentation broth over time (Yang et al., 2021).

Volatile fatty acids (VFAs) serve as the primary energy source for ruminant livestock, contributing to approximately 70–80% of the total digestive energy (Abbas et al., 2020; Chen et al., 2024). In the present study, an investigation into the *in vitro* fermentation of silage maize over varying fermentation durations revealed that, the treatment group exhibited a significant increase in the rumen fermentation index of acetate on day 30, followed by a marked decrease on day 60. Meanwhile, in terms of the three different fermentation days, the acetate index of the two groups on day 60 of fermentation were also significantly lower than that on day 0. This phenomenon may be attributed to the activity of acetic acid-producing bacteria during the initial 30 days, which stimulated a rapid proliferation of lactic acid-utilizing bacteria and fiber-degrading bacteria. These microbial populations facilitated the conversion of hydrogen and carbon dioxide into acetic acid. Additionally, the fiber-degrading bacteria enhanced nutrient fermentation, allowing for the complete utilization of acetic acid by rumen microorganisms, thereby participating in energy metabolism and fat synthesis, which ultimately resulted in the production of substantial amounts of acetic acid (Janssen, 2010). The significant increase in rumen acetic acid concentration suggests a shift toward an acetic acid-dominant fermentation type, which was advantageous for milk fat synthesis and consequently enhances both milk production and milk fat percentage (Urrutia and Harvatine, 2017). Conversely, in long-term silage aged more than 60 days, WSC was depleted, leading to the accumulation of lactic acid. In this phase, microorganisms shift their metabolism to lactic acid, which promotes the production of propionic and butyric acids, often associated with the proliferation of Megasphaera or Clostridium species (Dhakal et al., 2024). This is consistent with the results of this experiment in terms of the number of fermentation days. The significant elevation of butyric acid by the addition of the treatment group’s days 0 and 60 silage maize substrate to rumen fluid may be due to the ability of *Saccharomyces cerevisiae* to modulate the structure of the rumen microbial community and increase the number or activity of butyric acid-producing bacteria. Xue et al.’s research indicates that *Saccharomyces cerevisiae* can provide additional nutrients that promote microbial growth and metabolism, further increasing butyric acid production (Xue et al., 2022). The *in vitro* analysis of rumen VFA propionic acid content in the treatment group at 30 days of fermentation revealed a significant reduction, which may be associated with a decrease in pH levels. It has been demonstrated that propionic acid concentrations decline as rumen pH decreases. This phenomenon can be attributed to the varying sensitivities of the production pathways for different VFAs to pH fluctuations, with the production pathway for propionic acid being inhibited at lower pH levels (Liang et al., 2024). Wang et al. (2023) reported that a specific level of supplementation with *Saccharomyces cerevisiae* cultures may enhance the production of butyric and valeric acids while concurrently reducing the production of propionic acid, thereby mitigating the potential for toxicity to some extent. Total volatile acids, and butyric acids, exhibited a pattern of initial increase followed by a decrease and then a subsequent increase. Additionally, with the incorporation of silage substrate over the fermentation period, the A: P ratio demonstrated a similar trend of increase followed by decrease. This pattern may be attributed to the pre-fermentation phase of silage maize crude fiber, which is primarily linked to the production of acetic and butyric acids. Isobutyric and isovaleric acids, which are degradation products of branched-chain amino acids (e.g., valine and leucine), are rapidly acidified (pH < 4.5) upon the addition of silage. This acidification inhibits the proteolytic activity of spoilage bacteria in silage, thereby reducing the release of branched-chain fatty acids produced in the rumen (Agnihotri et al., 2022; Zhuang et al., 2024). The observed increase in total volatile acids further indicates that the degradation of dry matter in the rumen yields a greater quantity of fermentation substrates, which are subsequently utilized by microorganisms to produce additional VFAs. This observation can be elucidated by the increase in DMD noted in this experiment.

The dry matter degradation rate (DMD) serves as a critical index for assessing the efficacy of organic matter degradation during *in vitro* fermentation of forage. A higher DMD indicates a more effective degradation of organic matter, thereby enhancing the nutritional value of maize silage (Wang et al., 2013b). Increased gas production (GP) reflects a greater extent of feed degradation within the rumen, and total gas production (TGP) is typically proportional to DMD. This relationship was corroborated by the findings of the present experiment, which demonstrated a significant increase in both DMD and TGP at 30 days of fermentation. This enhancement may be attributed to the addition of the microbial additives, which stimulate the growth and reproduction of specific microorganisms in the rumen. Consequently, larger amounts of proteases, fiber-degrading enzymes, amino acids and vitamins were secreted. Simultaneously, substrate degradation released abundant carbohydrates usable by the host and provided growth factors and a beneficial bacterial flora essential for normal rumen fermentation (Onaleye et al., 2018; Zhang et al., 2020).

There are several limitations in the present study. At first, the scale of this study, involving two groups of 20 lactating cows, was somewhat limited. Furthermore, due to the limitations of the plateau conditions, it was impossible to collect rumen fluid. However, future trials will expand the number of animals to validate and extend the conclusions of this research. Secondly, since the *in vitro* fermentation collected from Xiangxi yellow cattle, these results have certain limitations, and its applicability to Xizang cattle needs further study.

## 5 Conclusion

Microbial additives enhanced crude protein, lactic acid, and acetic acid levels in whole-plant maize silage in Xizang, yielding higher sensory scores. Fermentation for 60 days improved milk protein content in late-lactation Tibetan Holstein cows, alongside increased *in vitro* total volatile fatty acids, dry matter degradation rates, and microbial protein content, thereby boosting ruminal digestibility and utilization of the silage. Furthermore, microbial additives maintain efficacy under low-temperature and low-oxygen partial pressure conditions, increasing the abundance of Firmicutes phylum and *Weissella* genus while reducing *Acetobacter* abundance. These measures facilitate a rapid decrease in silage pH and an increase in lactic acid content, thereby mitigating feed spoilage and enhancing feed quality. This is of great significance for the regional particularity and its guiding value for animal husbandry on the plateau. Future research will focus on large-scale dairy farms on the Xizang Plateau to expand the sample size of dairy cows for *in vivo* validation of silage. By integrating high-throughput sequencing and complementary technologies, the system will monitor the influence of microbial additives in silage on the dominant flora that provides core precursors for the synthesis of milk components during the rumen fermentation process in dairy cows after they consume silage, as well as the dynamic changes in rumen flora, and thereby verify the correlation with microbial additives.

## Data Availability

The datasets presented in this study can be found in online repositories. The names of the repository/repositories and accession number(s) can be found at: http://www.ncbi.nlm.nih.gov/bioproject/PRJNA1288161.

## Footnotes

1
https://www.genescloud.cn
